# Non-invasive detection of slow conduction with cardiac magnetic resonance imaging for ventricular tachycardia ablation

**DOI:** 10.1093/europace/euae025

**Published:** 2024-01-23

**Authors:** Sara Vázquez-Calvo, Judit Mas Casanovas, Paz Garre, Paula Sánchez-Somonte, Pasquale Valerio Falzone, Laura Uribe, Eduard Guasch, José Maria Tolosana, Roger Borras, Rosa M Figueras i Ventura, Elena Arbelo, José T Ortiz-Pérez, Susana Prats, Rosario J Perea, Josep Brugada, Lluís Mont, Andreu Porta-Sanchez, Ivo Roca-Luque

**Affiliations:** Institut Clinic Cardiovascular, Hospital Clínic, Universitat de Barcelona, Villarroel, 170, 08036 Barcelona, Spain; Institut d’Investigacions Biomèdiques August Pi i Sunyer (IDIBAPS), Barcelona, Spain; Institut Clinic Cardiovascular, Hospital Clínic, Universitat de Barcelona, Villarroel, 170, 08036 Barcelona, Spain; Institut Clinic Cardiovascular, Hospital Clínic, Universitat de Barcelona, Villarroel, 170, 08036 Barcelona, Spain; Institut d’Investigacions Biomèdiques August Pi i Sunyer (IDIBAPS), Barcelona, Spain; Institut Clinic Cardiovascular, Hospital Clínic, Universitat de Barcelona, Villarroel, 170, 08036 Barcelona, Spain; Institut d’Investigacions Biomèdiques August Pi i Sunyer (IDIBAPS), Barcelona, Spain; Institut Clinic Cardiovascular, Hospital Clínic, Universitat de Barcelona, Villarroel, 170, 08036 Barcelona, Spain; Institut d’Investigacions Biomèdiques August Pi i Sunyer (IDIBAPS), Barcelona, Spain; Institut Clinic Cardiovascular, Hospital Clínic, Universitat de Barcelona, Villarroel, 170, 08036 Barcelona, Spain; Institut d’Investigacions Biomèdiques August Pi i Sunyer (IDIBAPS), Barcelona, Spain; Institut Clinic Cardiovascular, Hospital Clínic, Universitat de Barcelona, Villarroel, 170, 08036 Barcelona, Spain; Institut d’Investigacions Biomèdiques August Pi i Sunyer (IDIBAPS), Barcelona, Spain; Centro de Investigación Biomédica en Red, Enfermedades Cardiovasculares (CIBERCV), Madrid, Spain; Institut Clinic Cardiovascular, Hospital Clínic, Universitat de Barcelona, Villarroel, 170, 08036 Barcelona, Spain; Institut d’Investigacions Biomèdiques August Pi i Sunyer (IDIBAPS), Barcelona, Spain; Centro de Investigación Biomédica en Red, Enfermedades Cardiovasculares (CIBERCV), Madrid, Spain; Institut Clinic Cardiovascular, Hospital Clínic, Universitat de Barcelona, Villarroel, 170, 08036 Barcelona, Spain; Institut d’Investigacions Biomèdiques August Pi i Sunyer (IDIBAPS), Barcelona, Spain; Centro de Investigación Biomédica en Red e Salud Mental, CIBERSAM, Instituto de Salud Carlos III, Madrid, Spain; ADAS 3D Medical S.L., Barcelona, Spain; Institut Clinic Cardiovascular, Hospital Clínic, Universitat de Barcelona, Villarroel, 170, 08036 Barcelona, Spain; Institut d’Investigacions Biomèdiques August Pi i Sunyer (IDIBAPS), Barcelona, Spain; Centro de Investigación Biomédica en Red, Enfermedades Cardiovasculares (CIBERCV), Madrid, Spain; Institut Clinic Cardiovascular, Hospital Clínic, Universitat de Barcelona, Villarroel, 170, 08036 Barcelona, Spain; Institut d’Investigacions Biomèdiques August Pi i Sunyer (IDIBAPS), Barcelona, Spain; Centro de Investigación Biomédica en Red, Enfermedades Cardiovasculares (CIBERCV), Madrid, Spain; Institut Clinic Cardiovascular, Hospital Clínic, Universitat de Barcelona, Villarroel, 170, 08036 Barcelona, Spain; Institut d’Investigacions Biomèdiques August Pi i Sunyer (IDIBAPS), Barcelona, Spain; Institut Clinic Cardiovascular, Hospital Clínic, Universitat de Barcelona, Villarroel, 170, 08036 Barcelona, Spain; Institut d’Investigacions Biomèdiques August Pi i Sunyer (IDIBAPS), Barcelona, Spain; Institut Clinic Cardiovascular, Hospital Clínic, Universitat de Barcelona, Villarroel, 170, 08036 Barcelona, Spain; Institut d’Investigacions Biomèdiques August Pi i Sunyer (IDIBAPS), Barcelona, Spain; Centro de Investigación Biomédica en Red, Enfermedades Cardiovasculares (CIBERCV), Madrid, Spain; Institut Clinic Cardiovascular, Hospital Clínic, Universitat de Barcelona, Villarroel, 170, 08036 Barcelona, Spain; Institut d’Investigacions Biomèdiques August Pi i Sunyer (IDIBAPS), Barcelona, Spain; Centro de Investigación Biomédica en Red, Enfermedades Cardiovasculares (CIBERCV), Madrid, Spain; Institut Clinic Cardiovascular, Hospital Clínic, Universitat de Barcelona, Villarroel, 170, 08036 Barcelona, Spain; Institut d’Investigacions Biomèdiques August Pi i Sunyer (IDIBAPS), Barcelona, Spain; Institut Clinic Cardiovascular, Hospital Clínic, Universitat de Barcelona, Villarroel, 170, 08036 Barcelona, Spain; Institut d’Investigacions Biomèdiques August Pi i Sunyer (IDIBAPS), Barcelona, Spain; Centro de Investigación Biomédica en Red, Enfermedades Cardiovasculares (CIBERCV), Madrid, Spain

**Keywords:** Ventricular tachycardia ablation, Cardiac magnetic resonance, Conducting channels, Isochronal late activation maps, Deceleration zones

## Abstract

**Aims:**

Non-invasive myocardial scar characterization with cardiac magnetic resonance (CMR) has been shown to accurately identify conduction channels and can be an important aid for ventricular tachycardia (VT) ablation. A new mapping method based on targeting deceleration zones (DZs) has become one of the most commonly used strategies for VT ablation procedures. The aim of the study was to analyse the capability of CMR to identify DZs and to find predictors of arrhythmogenicity in CMR channels.

**Methods and results:**

Forty-four consecutive patients with structural heart disease and VT undergoing ablation after CMR at a single centre (October 2018 to July 2021) were included (mean age, 64.8 ± 11.6 years; 95.5% male; 70.5% with ischaemic heart disease; a mean ejection fraction of 32.3 ± 7.8%). The characteristics of CMR channels were analysed, and correlations with DZs detected during isochronal late activation mapping in both baseline maps and remaps were determined. Overall, 109 automatically detected CMR channels were analysed (2.48 ± 1.15 per patient; length, 57.91 ± 63.07 mm; conducting channel mass, 2.06 ± 2.67 g; *protectedness*, 21.44 ± 25.39 mm). Overall, 76.1% of CMR channels were associated with a DZ. A univariate analysis showed that channels associated with DZs were longer [67.81 ± 68.45 vs. 26.31 ± 21.25 mm, odds ratio (OR) 1.03, *P* = 0.010], with a higher border zone (BZ) mass (2.41 ± 2.91 vs. 0.87 ± 0.86 g, OR 2.46, *P* = 0.011) and greater protectedness (24.97 ± 27.72 vs. 10.19 ± 9.52 mm, OR 1.08, *P* = 0.021).

**Conclusion:**

Non-invasive detection of targets for VT ablation is possible with CMR. Deceleration zones found during electroanatomical mapping accurately correlate with CMR channels, especially those with increased length, BZ mass, and protectedness.

## Graphical Abstract

**Graphical Abstract:**
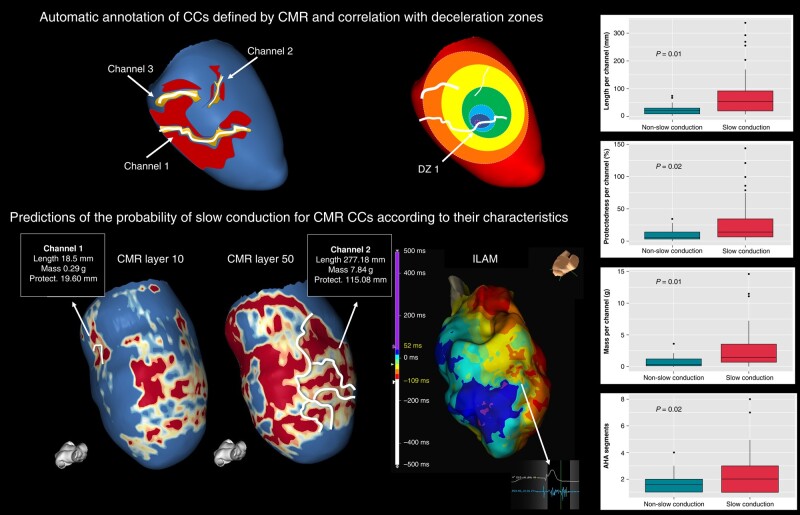

**What’s new?**

- This is the first study to our knowledge that analyses not only the relationship of cardiac magnetic resonance (CMR) channels with deceleration zones (DZs) but also the predictors of arrhythmogenicity.
- More than 75% of the channels of CMR were related to DZs.
- Some characteristics of the CMR channel were strongly related to arrhythmogenicity (in terms of the presence of DZ) as the length (67.81 vs. 26.31 mm, *P* = 0.01) and mass of the border zone within the channel (2.41 vs. 0.87 g, *P* = 0.01).
- These findings could have immediate clinical applications to refine CMR-aided ventricular tachycardia (VT) substrate ablation (to ablate mainly the channels with certain characteristics, thereby shortening the ablation procedures). In addition, if confirmed by other studies, the findings could also have implications for improving the specificity of CMR to stratify the risk of VT in different populations.

## Introduction

Substrate-based radiofrequency catheter ablation has become a standard procedure for the treatment of scar-related ventricular tachycardia (VT).^1^ The main mechanism behind scar-related VT is the re-entrant circuit. This circuit is caused by the presence of a slowly conducting area or channel (CC) usually within the border zone (BZ) tissue surrounded by the scar and connecting healthy tissue. Several strategies have been developed to identify these slow conduction areas using electroanatomical maps (EAMs) during ablation.^2^ Substrate mapping focused on the analysis of abnormal electrograms after main ventricular activation—so-called late potentials (LPs)—or local abnormal ventricular activity (LAVA) within low-voltage regions is currently the principal method to define the arrhythmic substrate during stable rhythm when VT activation mapping is not possible.^3^ An additional mapping tool for the detection of slow conduction areas, called isochronal late activation mapping (ILAM), was described by Irie *et al*.^4^ and has recently been shown to be very specific for the VT critical zone.^5^

From a structural point of view, late gadolinium enhancement-cardiac magnetic resonance (LGE-CMR) has demonstrated to precisely identify and characterize the arrhythmogenic substrate, being able to depict the CCs with a high correlation to the EAMs^6,7^ even in patients with an implantable cardioverter defibrillator (ICD) *in situ* with a dedicated LGE sequence [wideband (WB) sequence].^8^ With regard to VT ablation procedures, LGE-CMR is widely used as a standard method, since it can greatly facilitate pre-procedural planning and procedural success.^9,10^

In this study, to continue improving the capability of CMR to define the arrhythmic substrate, we studied the correlation between CMR conducting channels and deceleration zones (DZs) detected during ILAM with a specific analysis of CMR channel characteristics related to the presence of DZs as a marker of the arrhythmogenicity of conducting channels.

## Methods

### Study population

This was a prospective observational study of all consecutive patients with structural heart disease who underwent VT ablation in a single centre (Hospital Clinic, University of Barcelona) from October 2018 to July 2021. Late gadolinium enhancement-cardiac magnetic resonance was performed within 6–12 months before ablation. The exclusion criterion of the study was the absence of pre-procedural CMR. All patients provided written informed consent. The study was carried out according to the Declaration of Helsinki guidelines and the deontological code of our institution. The study protocol was approved by the ethics committee of the hospital.

### Pre-procedural cardiac magnetic resonance

A 3 T scan was performed in patients without an ICD, and a 1.5 T scan with a WB sequence was used in patients with an ICD to reduce image artefacts, as described previously.^8^ The analysis of the arrhythmic substrate was performed using ADAS 3D software (ADAS 3D, Galgo Medical S.L.) following a standardized and widely validated protocol.^10^ In brief, a semiautomatic segmentation of the left ventricle was performed by an expert operator. Next, ADAS 3D divided the myocardium thickness into a total of nine three-dimensional (3D) maps from the endocardium to the epicardium. The LGE pixel signal intensity maps obtained from the CMR scans were projected onto each layer with a trilinear interpolation algorithm and were colour-coded using 40 ± 5 and 60 ± 5% of the maximum intensity as thresholds. Therefore, areas with high LGE (≥60%) were coded as dense scar tissue and coloured red; healthy tissue without LGE (≤40%) was coloured blue; and BZs, identified as areas with an intermediate percentage of LGE (between 40 ± 5 and 60 ± 5%), were coloured in an intermediate colour range from yellow to green. According to the LGE distribution from 10% (the layer closest to the endocardium) to 90% (the layer closest to the epicardium), the substrate was defined as endocardial when LGE affected 10–30% of the layer, epicardial when LGE affected 60% of the outer layer, and mid-mural when LGE was confined to the internal layer of myocardial thickness without endocardial or epicardial distribution. Areas of LGE >75% in myocardial thickness were considered transmural.

Channels and their characteristics were analysed automatically by the system. *Protectedness*, which is a parameter based on the length of the channel covered (‘protected’) by dense scar tissue and known to correlate with VT critical areas,^11^ was also calculated. To determine protectedness, the percentage of the CMR channel perimeter containing core tissue, healthy tissue, or BZ tissue within a 3.5 mm radius was examined. Local protectedness was then determined based on these percentages: if healthy tissue was present, local protection was set to zero; if no healthy tissue was found, protection depended on the percentage of the perimeter coinciding with core tissue. A point with <15% core had 0% local protection (fully unprotected), while a point with >40% had 100% local protection (fully protected). Core values between 15 and 40% were linearly mapped to local protection values between 0 and 100%. The local protection values were integrated over the entire CMR channel.

### High-density mapping: substrate and isochronal late activation mapping

Procedures were performed under general anaesthesia. Access to the left ventricle was achieved with a transseptal and/or retrograde aortic approach at the discretion of the operator. Epicardial mapping was performed in cases when an epicardial origin of VT was suspected and in cases of previously failed endocardial ablation.

The mapping approach consisted of performing ILAM of the left ventricle during right ventricular pacing with a stable cycle length of 600 ms using an HDGrid catheter and EnSite Precision system (Abbott Medical, USA). These maps were executed by annotating the latest deflection of the electrograms (EGMs) (Last Deflection algorithm, Abbott Medical) of HDGrid orthogonal signals (HD Wave algorithm, Abbot Medical) and dividing the whole activation of the chamber into eight equally distributed isochrones, with white being the earliest and purple being the latest isochrone. Deceleration zones were defined as regions with isochronal crowding, with >3 isochrones within a 1 cm radius, as previously described.^4^ Late gadolinium enhancement-cardiac magnetic resonance post-processed images were carefully and systematically aligned according to the AP and LL projections in the mapping system and CMR. They were then visualized into the navigation system side by side with the EAMs.

### Ablation strategy and remapping

After an analysis of the generated maps, the tagged EGMs, and the channels defined by CMR, the HDGrid catheter was positioned in the potential VT isthmus (DZ with higher isochronal crowding, especially if correlated with a channel by CMR). Ventricular tachycardia was induced by programmed electrical stimulation,^12^ and if it was haemodynamically tolerated, activation mapping for diastolic and pre-systolic activity was performed. In cases in which VT was not haemodynamically tolerated, the VT critical side was defined as the area with a fast transition from good pace mapping (suggesting VT exit site) to poor pace mapping (suggesting VT entrance site). In these cases, the initial target for ablation was the central VT isthmus where diastolic EGMs were observed. After the critical areas of induced VT were targeted, substrate ablation was performed by strictly targeting the rest of the DZs. Radiofrequency was delivered using an externally irrigated 3.5 mm tip ablation catheter with 45°C temperature control (at an irrigation rate of 26–30 mL/min), at a power limit of 40–50 W. Remapping with the HDGrid catheter was performed to assess the abolition of the DZs and/or detect new DZs, especially in areas of interest according to LGE-CMR. Additional radiofrecuency (RF) lesions were delivered until complete elimination of the DZs. Acute success was defined as total abolition of DZs as well as the lack of VT inducibility at the end of the procedure.

### *Post hoc* offline analysis

**Figure 1:**
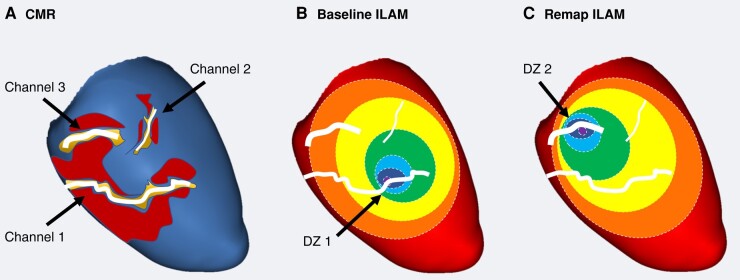
(*A*) A schematic of an automatic annotation of CCs defined by CMR. Different lengths, widths, and levels of protectedness can be observed for each channel. (*B*) The correlation with DZs was annotated in every map. In this case, Channel 1 is associated with a DZ in the baseline map, with Channel 3 correlated with a new DZ observed in the remap. (*C*) Channel 2 is not related to any DZ in this example. CC, conducting channel; CMR, cardiac magnetic resonance; DZ, deceleration zone; ILAM, isochronal late activation mapping.

The detection and analysis of CMR channels was performed automatically by ADAS 3D software, as described in the Methods section. This analysis was done before the ablation procedure and therefore without any information related to EAM. A *post hoc* analysis of the DZs was done by an experienced operator of all maps (basal maps and remaps), blinded to CMR data. Finally, a visual correlation between each channel and the DZs was performed, not only with those observed in the baseline map but also with those observed in the remaps (*Figure 1*).

### Clinical follow-up

All patients were followed routinely during a 1-year follow-up with clinical visits at 1, 6, and 12 months and with remote device monitoring when possible. Ventricular tachycardia recurrence was defined as any sustained VT episode or sudden death.

### Statistical analysis

Continuous variables are presented as the mean value ± standard deviation, and minimum and maximum values were compared by using Student’s *t*-test. Categorical variables are expressed as total numbers (percentages) and were compared between groups using the *χ*^2^ test. A generalized linear mixed-effects model for binary response was used to study the effects of LGE-CMR characterization to predict slow conduction areas. Odds ratios and 95% confidence limits were calculated. A two-sided Type I error of 5% was used for all tests. A statistical analysis was performed using R software for Windows version 4.2.2 (R Project for Statistical Computing; Vienna, Austria).

## Results

### Study population

Between October 2018 and July 2021, 58 patients underwent ablation procedures for scar-related VT at our institution. Twelve patients were excluded due to the inability to perform CMR (arrhythmic storm, claustrophobia, etc.), and two were excluded due to poor-quality CMR. Overall, 44 patients were included in the study. The median age was 64.8 ± 11.6 years, 95.5% were male, and the median ejection fraction was 32.3 ± 7.8%. A total of 70.5% had ischaemic cardiomyopathy, and 15.9% presented with a VT storm. The clinical characteristics are detailed in *Table 1*. In 73.7% of patients, 1.5 T CMR with a WB sequence was performed.

**Table 1:** Clinical characteristics

	Patients studied (*n* = 44)
Age (years)	64.8 ± 11.6 (26–82)
Male sex	42 (95.5%)
Hypertension	28 (63.6%)
Diabetes	20 (46.5%)
Dyslipidaemia	27 (62.8%)
COPD	6 (15.0%)
CKD	11 (28.9%)
NYHA class	
I	7 (20.6%)
II	23 (67.6%)
III and IV	4 (11.8%)
Ischaemic cardiomyopathy	31 (70.5%)
Permanent AF	10 (23.3)
ACEIs	23 (60.5%)
Beta-blockers therapy	29 (76.3%)
Sotalol therapy	4 (10.5%)
Amiodarone therapy	31 (81.6%)
VT storm	7 (15.9%)
LVEF (%)	32.3 ± 7.8
LVEDD (mm)	61.4 ± 10.2

AF, atrial fibrillation; CKD, chronic kidney disease; COPD, chronic obstructive pulmonary disease; ACEIs, angiotensin-converting enzyme inhibitors; LVEF, left ventricular ejection fraction; LVEDD, left ventricular end-diastolic diameter; NYHA, New York Heart Association; VT, ventricular tachycardia.

### Procedural characteristics

An endocardial procedure was performed in all patients, with a transeptal approach in the majority of patients (97.6%) combined with retro-aortic access in 63.6% of patients. An epicardial procedure was performed in three (6.8%) patients. During the basal electrophysiological study, 83.3% of patients were inducible for VT, with a median of 1.71 ± 1.55 VTs induced per patient (*Table 2*).

**Table 2:** Procedural characteristics

	*n* = 44
Endocardial approach	40 (93.0%)
Endoepicardial approach	3 (7.0%)
Arterial access	26 (63.4%)
Transeptal access	41 (97.6%)
Agilis	24 (60.0%)
Number of mapping points	2199.75 ± 703.64
Number of VT inductions	1.71 ± 1.55
Number of targeted VTs	1.62 ± 1.55
Procedural time (min)	237.72 ± 48.56
X-ray time	37.97 ± 12.36
Number of RF applications	57.46 ± 24.44
RF time (s)	1883.38 ± 848.80
Final non-inducibility	33 (82.5%)
Absence of residual slow conduction	33 (82.5%)

VT, ventricular tachycardia.

### Late gadolinium enhancement-cardiac magnetic resonance characteristics and predictors of conducting channel arrhythmogenicity

A total of 109 CMR channels were studied (2.48 ± 1.15 channels per patient). The majority of channels had an endocardial location (34.9%) or transmural location (22.0%). The median length per channel was 57.91 ± 63.07 mm, with a median mass of 2.06 ± 2.67 g. The median protectedness per channel was 21.44 ± 25.39 mm.

Overall, 76.1% of CMR channels were associated with a DZ during the procedure: 64 CMR channels (58.7%) were correlated with DZs observed in the baseline map, and 19 CMR channels (22.9%) were associated with DZs not observed in the initial map but detected during the remap after the first ablation set. Additionally, 41.8% of CMR channels correlated with a mapped VT critical site. No differences were observed between ischaemic and non-ischaemic patients in terms of the correlation between CMR channels and slow conduction areas (74.49 vs. 70.96%, *P* = 0.70).

**Figure 2:**
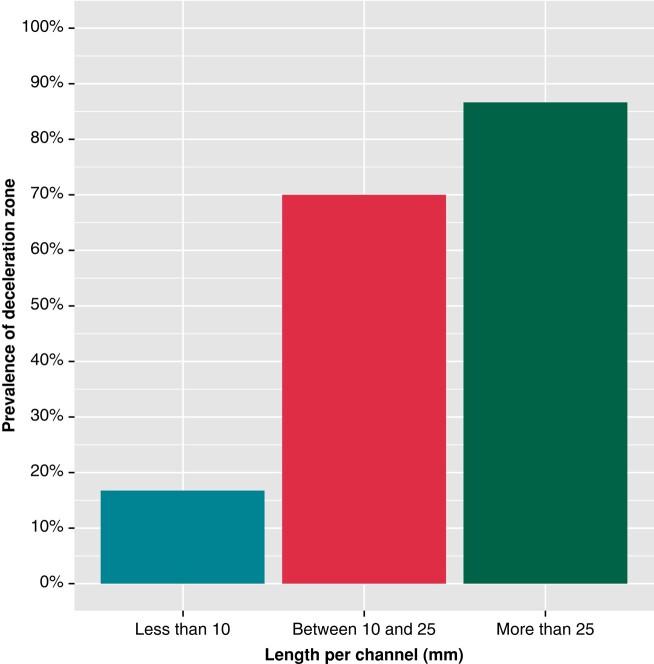
Probability of an association between CMR channels and DZs according to the length of the channel. CCs with a length <10 mm have a probability rate of only 16.7% to detect a DZ. In contrast, CCs that are longer than 25 mm have a probability rate as high as 86.7%. CC, conducting channel; CMR, cardiac magnetic resonance; DZ, deceleration zone.

**Figure 3:**
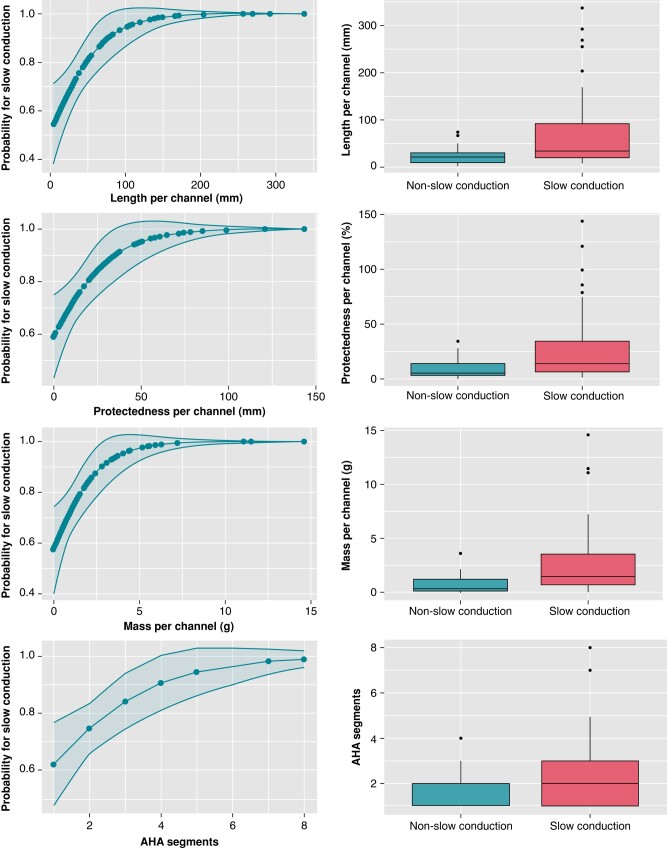
Predictions of the probability of slow conduction with logistic regression for length, mass, protectedness, and cumulative number of affected American Heart Association (AHA) segments.

The univariate analysis showed that channels associated with DZs were longer [67.81 ± 68.45 vs. 26.31 ± 21.25 mm, odds ratio (OR) 1.03, *P* = 0.01], with a higher mass (2.41 ± 2.91 vs. 0.87 ± 0.86 g, OR 2.46, *P* = 0.01) and greater protectedness (24.97 ± 27.72 vs. 10.19 ± 9.52 mm, OR 1.08, *P* = 0.02) compared with those not related to DZs. Regarding length and its correlation with DZs, channels over 25 mm had a probability of 86.7% (standard error [SE] 0.04) vs. only 16.7% (SE 0.15) for channels <10 mm (*Figure 2*). Other highly correlated parameters, such as the number of AHA segments or the number of layers affected, were also significantly higher in CMR channels associated with DZs (2.48 ± 1.47 vs. 1.77 ± 0.82, OR 2.09, *P* = 0.02 and 3.43 ± 2.50 vs. 2.31 ± 1.62, OR 1.44, *P* = 0.02). Channels with transmural involvement were also more likely to be associated with slow conduction areas (OR 6.42, *P* = 0.04). Cardiac magnetic resonance channel width was not a predictor of arrhythmogenicity in our study. No differences were observed between ischaemic and non-ischaemic patients. In *Table 3* and *Figure 3*, the relationship between CMR channel parameters and DZs is shown.

**Table 3:** A univariate analysis of channel characteristics and the primary endpoint (slow conduction properties)

	Total (*n* = 109)	Slow conduction (*n* = 83)	Non-slow conduction (*n* = 26)	OR	*P*-value
Length per channel (mm)	57.91 ± 63.07 (4.16–338.10)	67.81 ± 68.45	26.31 ± 21.25	1.03 (1.01–1.06)	**0**.**01**
Mass per channel (g)	2.06 ± 2.67 (0.01–14.59)	2.41 ± 2.91	0.87 ± 0.86	2.46 (1.23–4.92)	**0**.**01**
Width per channel (mm)	5.02 ± 1.82 (0.01–9.34)	5.23 ± 1.51	4.38 ± 2.50	1.29 (0.90–1.67)	0.06
Protectedness per channel (mm)	21.44 ± 25.39 (0.01–143.51)	24.97 ± 27.72	10.19 ± 9.52	1.08 (1.01–1.2)	**0**.**02**
Layers affected by CMR channels					
Exclusively endocardium	38 (34.9%)	25	13	0.35 (0.11–1.07)	0.06
Exclusively mesocardium	2 (1.8%)	2	0	NA	NA
Exclusively epicardium	18 (16.5%)	15	3	1.63 (0.40–6.72)	0.50
Exclusively endo/meso	20 (18.3%)	13	7	0.56 (0.18–1.78)	0.32
Exclusively meso/epi	7 (6.4%)	6	1	NA	NA
Transmural	24 (22.0%)	22	2	6.42 (1.11–37.28)	**0.04**
Number of layers per channel	3.17 ± 2.36 (1–9)	3.43 ± 2.50	2.31 ± 1.62	1.44 (1.01–1.95)	**0**.**02**
Number of AHA segments per channel	2.30 ± 1.37 (1–8)	2.48 ± 1.47	1.77 ± 0.82	2.09 (1.13–3.86)	**0**.**02**

CMR, cardiac magnetic resonance; OR, odds ratio.

Bold values denote statistical significance (*p* < 0.05).

### Radiofrequency delivery, acute success, and follow-up

The median duration of RF application was 37.97 ± 12.36 min, with a total procedural time of 237.72 ± 48.56 min. Non-inducibility at the end of the procedure was achieved in 82.5% of patients, and total elimination of DZs/LAVA/LPs was achieved in 82.5%.

Complications were observed in three patients: two patients presented cardiac tamponade, and one patient suffered an intractable VT storm during mapping of the left ventricle complicated with cardiorespiratory arrest that required advanced cardiopulmonary resuscitation manoeuvres.

After 1 year of follow-up, three patients died due to non-arrhythmic-related events. Ventricular tachycardia recurrence after 1 year of follow-up was 21.95% (19.35% in ischaemic cardiomyopathy patients and 30% in non-ischaemic cardiomyopathy patients) and 25.0% after 2 years of follow-up.

## Discussion

The main findings of our study are as follows. First, three out of four CMR-detected CCs colocalized with DZs during high-density EAM. Second, CMR channels with increased length, BZ mass, and protectedness had a higher correlation with slow conduction areas.

Tissue characterization with CMR imaging using late gadolinium enhancement has evolved in recent years, and it is currently the most commonly used non-invasive tool to assess the arrhythmic substrate in patients with structural heart disease. The use of gadolinium allows us to distinguish not only the dense scar from the healthy tissue but also the BZ where the CC is located, which in turn generates the reentrant circuit and potentially maintains the VT.

Previous works have shown a high spatial correlation between CMR channels and voltage channels obtained during VT substrate mapping procedures.^13^ However, these voltage channels have low specificity to detect VT isthmuses.^14^ Notably, ablation strategies based exclusively on targeting the channel responsible for the induced/clinical VT have failed, resulting in worse clinical rates of VT recurrence in the follow-up period compared with more extensive substrate-ablation methods. One potential explanation relies on the capability of the myocardium tissue to change its properties and, therefore, its potential arrhythmogenicity according to pacing cycle length, site of pacing, etc.,^15,16^ so different CCs could act as VT circuits or not under different situations. This could explain why targeting only the induced VT is not enough to avoid future VTs. For this reason, in recent years, substrate-ablation strategies have included not only ablation of the observed active VT circuit but also more extensive approaches. Several techniques have been proposed. One method based on a more detailed definition of the arrhythmic substrate is used to eliminate every LP or LAVA or even all scars (scar homogenization).^17,18^ Other authors have worked on a more functional concept of arrhythmogenicity, identifying and targeting pathological EGMs unmasked after extrastimulus^19–21^ or with decremental properties.^22,23^ One of the more recent and successful strategies is based on ILAM,^4,5^ in which the entire substrate is annotated considering not only its properties but also its relation to the surrounding tissue, defining DZs. These new strategies have been shown to improve VT ablation outcomes.

To date, CMR channels have demonstrated a great correlation with voltage channels, although the specificity of both for VT isthmuses detected during invasive VT mapping is low.^14^ Recently, our group published a study analysing the evolution of the DZ during VT ablation that also showed a high correlation between DZs and CMR channels (93.68%) in both the baseline ILAM and remaps.^24^ Interestingly, remapping allowed us to unmask DZs not observed in the baseline maps that were correlated with CMR channels. Our study is, to the best of our knowledge, the first to quantify and analyse the role of CMR in correctly identifying areas of slow conduction during substrate mapping performed with the ILAM technique.

### Applications of cardiac magnetic resonance to evaluate arrhythmogenicity

In our study, 76.1% of CMR channels were associated with DZs in the sinus- or right ventricle (RV)-paced map. Interestingly, 22.9% of all CMR channels were related to a DZ not observed in the baseline map but detected in the remap, which highlights the potential advantage of CMR to identify arrhythmic substrates compared with a static conventional map. Two possible hypotheses could explain this phenomenon: First, the methodology of ILAM could be very helpful in identifying the primary (or more evident) DZ, especially when *very LPs* are present in the area, but not a second or third DZ, even if abnormal EGMs are present. The explanation relies on the fact that the ILAM methodology divides the total activation time into eight isochrones. In this sense, when very LPs occur, the total time activation window will be very long, so each isochrone will last a substantial amount of time, grouping isochrones with less delayed potentials and dissipating some potential DZs. After the ‘primary’ DZ is ablated, the ‘secondary’ DZs can therefore be clearly observed.

**Figure 4:**
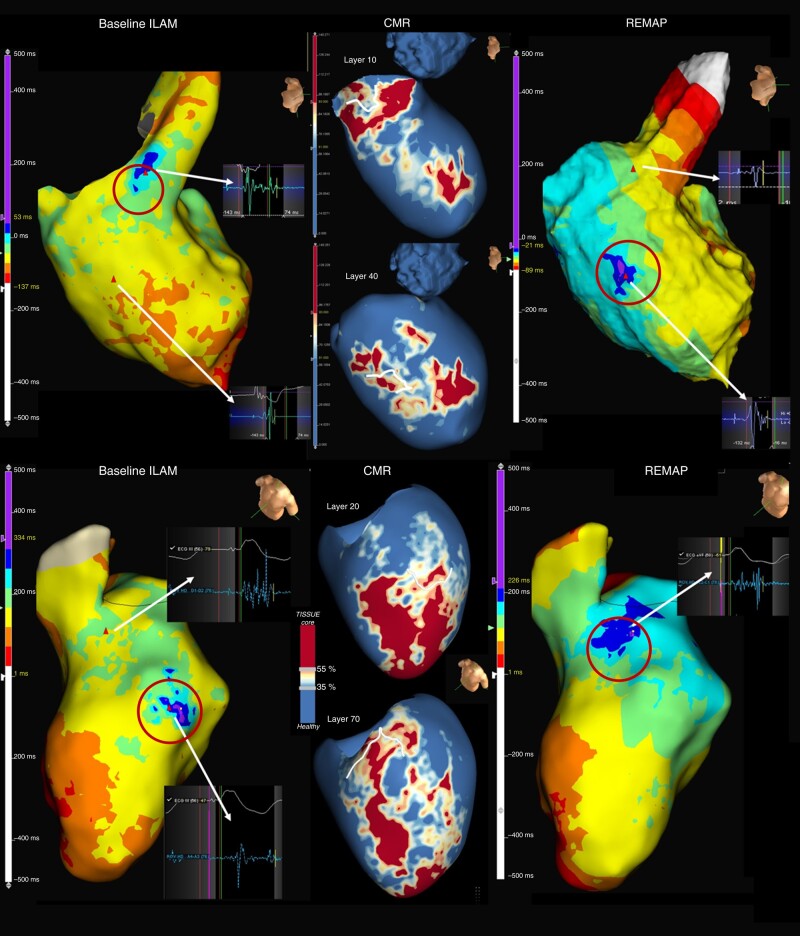
Examples of ILAM and CMR of two patients. In the upper panel, a basal anteroseptal endocardium channel correlated with a DZ in the baseline map. The remap shows how the primary DZ is eliminated, but a new DZ appears (not present in the baseline map), which is also correlated with CMR channels. The lower panel shows the mid-ventricle epicardial channel within the scar correlated with a DZ in the endocardial map. After ablating this primary DZ, a second DZ is shown. This area already showed LAVA in the initial map, but because of the ILAM method, it was not initially detected, and only after ablation of the DZ with the latest EGM was LAVA seen in the ILAM. Interestingly, this new DZ also correlated with CMR channels (DZs are shown in circles). CMR, cardiac magnetic resonance; DZ, deceleration zone; ILAM, isochronal late activation mapping; LAVA, local abnormal ventricular activity.

Second, as we have previously explained, the properties of the myocardium can change in different circumstances, so different DZs can be expected with different cycles, or even after changing the electrical properties of the tissue by the use of radiofrequency. *Figure 4* shows an example of these proposed mechanisms. To confirm this hypothesis, it would be interesting to perform manoeuvres during the baseline map to unmask slow conduction (for example, with extrastimulus) in those CMR channels that are initially not apparent to determine if that area had slow conduction properties that were hidden at baseline.

Improving the capability of CMR to define arrhythmogenicity can lead to a myriad of applications, from guiding faster and more effective VT ablation procedures to creating a score based on scar characteristics to predict arrhythmic events in patients with structural heart disease.^25^ This last point could be particularly relevant since several studies have already shown the incremental value of scar characteristics as depicted by CMR to predict arrhythmic events when compared with ejection fraction.^26,27^ Indeed, a previous study from our group^28^ in 2021 identified the absence of channels as a variable with a high negative predictive value of VT events in patients with primary prevention, suggesting that those with <10 g of scar tissue and without CCs were at very low risk of having appropriate therapies (5.26 vs. 25.31% per year, *P* = 0.034).

On the other hand, VT ablation guided by CMR has been widely developed by Berruezo *et al*. Initially, the scar dechanneling technique^17^ suggested, in a non-randomized trial, that ablation of CCs based exclusively on CMR was effective with a lower need for RF delivery, higher non-inducibility rates after substrate ablation, and a higher VT recurrence-free survival.^10^ However, on the one hand, it has been shown in our study that not all CMR channels have arrhythmogenic properties. On the other hand, basal EAM did not contain all the relevant information about arrhythmogenicity, due to the influence of the electrode size and interelectrode spacing and angle of the incoming wavefront on the mapping catheter^29,30^ and due to the functional component of the substrate.^19,20,22^ In this sense, electrophysiologica manoeuvres to unmask slow conduction could be useful in areas where LGE-CMR shows CCs, but no suspicious EGMs are seen in basal EAM. Therefore, the combination of EAM and CMR could be the best strategy to determine ablation targets. Defining which characteristics of the CMR channels turn them into arrhythmogenic channels is a priority to increase the use of CMR with all its potential applications.

### Characteristics of the cardiac magnetic resonance conducting channels showing arrhythmic properties

**Figure 5:**
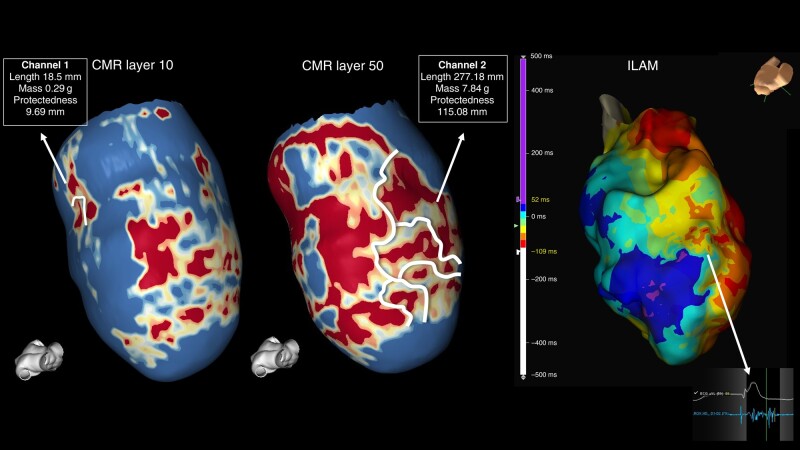
Cardiac magnetic resonance showing two different channels: Channel 1 located exclusively in the first endocardial layer with a shorter length, mass, and protectedness. Channel 2 observed in nine out of nine layers (transmural). ILAM shows a wide area with slow conduction properties correlated with Channel 2. CMR, cardiac magnetic resonance; ILAM, isochronal late activation mapping.

**Figure 6:**
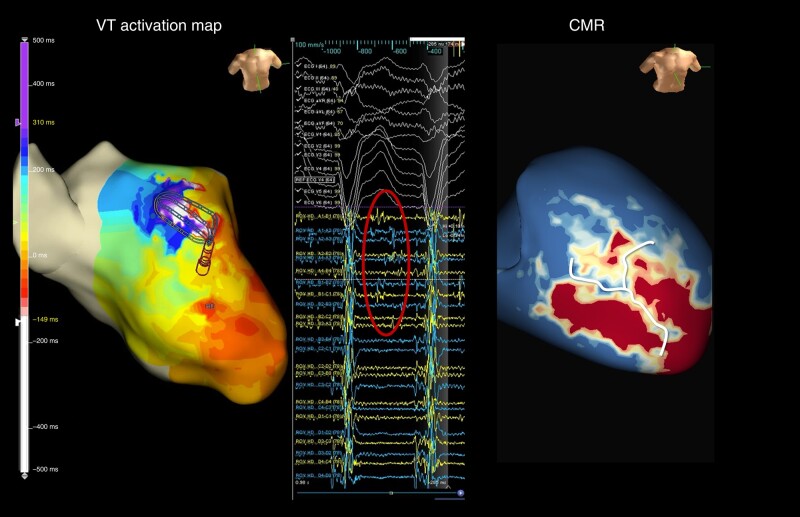
A ventricular tachycardia activation map showing an anterior VT circuit (left panel) with diastolic electrograms (circle) correlating with a long CMR channel (right panel). CMR, cardiac magnetic resonance; VT, ventricular tachycardia.

Our work attempts to characterize CMR channels to define which channels present arrhythmic properties, showing that those with greater length, mass, and protectedness are more likely to be correlated with DZs. Aziz *et al*.^5^ show that DZs had a higher correlation with the VT isthmus and were thus able to identify CMR channels that potentially correlated with these slow conduction areas before the VT ablation procedure, which could have an important role in substrate VT ablation. In addition, these results are consistent with those of Sanchez-Somonte *et al*.^11^ who analysed the characteristics of CMR channels associated with VT circuits (during VT activation maps, not during sinus- or RV-paced maps) and showed a greater probability of CCs correlated with VT with the same features as we have described (*Figures 5* and *6*).

Overall, our findings confirm the usefulness of CMR to understand 3D arrhythmic substrates. Characteristics of the CMR channels, such as length, mass, and protectedness, could become useful parameters to predict arrhythmic risk and to aid VT ablation, especially with its ability to identify potential areas of slow conduction not seen with conventional maps or only seen after ablation of the first slow conduction areas.

### Limitations

One potential limitation of this study is that the correlation between CMR channels and DZs was performed side by side and not merged. The merging process with Ensite Precision is technically challenging because it causes an artificial deformation of the cardiac anatomy. In addition, the merging process is very important in analysing quantitative data. However, for this study, only the correlation was assessed. In this sense, we believe that a side-by-side comparison of EAM and CMR images is an appropriate and reliable method and reproducible in clinical practice. On the other hand, 2 out of 46 CMRs were not analysed in our study due to bad quality, which is a reasonable percentage. Nevertheless, this high percentage in good-quality wideband CMR could be not extrapolated in centres where personnel had less experience.

Another limitation is that most of our cases were performed exclusively through endocardium access, and therefore, the epicardium surface was not mapped. This is attributed to the obvious safety concerns of a systematic endoepicardial approach. This could lead to an underestimation of the total number of detected DZs and, consequently, of the number of channels with slow conduction properties.

## Conclusions

Cardiac magnetic resonance–detected conducting channels accurately colocalize with DZs, especially those with a greater length, mass, and protectedness. These findings could illustrate the capability of CMR to depict the arrhythmogenic substrate, leading to multiple potential and relevant clinical applications.

## Data Availability

The authors declare that the data that support the findings of this study are available from the corresponding author.
